# Targeting programmed cell death via active ingredients from natural plants: a promising approach to cancer therapy

**DOI:** 10.3389/fphar.2024.1491802

**Published:** 2024-11-01

**Authors:** Qian Li, Yan Tong, Jianxiang Chen, Tian Xie

**Affiliations:** ^1^ School of Pharmacy and Department of Hepatology, The Affiliated Hospital of Hangzhou Normal University, Hangzhou Normal University, Hangzhou, China; ^2^ Key Laboratory of Elemene Class Anti-Cancer Chinese Medicines, Engineering Laboratory of Development and Application of Traditional Chinese Medicines, Collaborative Innovation Center of Traditional Chinese Medicines of Zhejiang Province, Hangzhou Normal University, Hangzhou, Zhejiang, China

**Keywords:** cancer therapy, plant natural compound, programmed cell death, pharmacology, signaling pathways

## Abstract

Cancer is a serious public health problem in humans, and prevention and control strategies are still necessary. Therefore, the development of new therapeutic drugs is urgently needed. Targeting programmed cell death, particularly via the induction of cancer cell apoptosis, is one of the cancer treatment approaches employed. Recently, an increasing number of studies have shown that compounds from natural plants can target programmed cell death and kill cancer cells, laying the groundwork for use in future anticancer treatments. In this review, we focus on the latest research progress on the role and mechanism of natural plant active ingredients in different forms of programmed cell death, such as apoptosis, autophagy, necroptosis, ferroptosis, and pyroptosis, to provide a strong theoretical basis for the clinical development of antitumor drugs.

## 1 Introduction

Cancers are the leading cause of death worldwide, with an estimated 20 million new cases and 9.7 million deaths in 2022, among which the most common types are breast, lung, colorectal, and liver cancers (Bray et al., 2024). At present, the mainstream approach to cancer treatment is surgery, supplemented by drug therapy (Aquina et al., 2021). However, surgery may induce the spread of cancer cells and cause ischemia or reperfusion injury, acute and chronic pain, and large wounds (Siddiqi and Johnston, 2023; Chen et al., 2019; Jacobs et al., 2020). Moreover, among the currently approved cytostatic drugs, many have relatively low specificity for tumors and are highly toxic (Schirrmacher, 2019). Therefore, the development of new treatments to improve the prognosis of cancer patients is urgently needed.

Natural plant products have always been an important source of new drugs. In ancient times, people used herbs and formulations as medicines to treat various diseases. In 1806, Friedrich Wilhelm Sertürner separated morphine from poppy, marking the beginning of the search for active ingredients from natural plants in modern times (Klockgether-Radke, 2002). Since then, many natural compounds have been isolated from plants, such as artemisinin, quinine and atropine, and these products and their structural analogues have historically made significant contributions to disease treatment (Zhang L. et al., 2020). Recently, natural products from plants, such as curcumin for treating pancreatic cancer and paclitaxel for treating lung cancer, breast cancer and other cancers, have played increasingly important roles in the treatment of malignant tumors. Furthermore, these products can be used as sensitizers to enhance the anticancer effects of chemotherapy drugs and radiotherapy and to reduce cancer resistance (Lin et al., 2020). Traditional Chinese medicines such as Radix Astragali, Baizhu, and Poria have been found to be effective in treating lung cancer when combined with EGFR inhibitors (Sui et al., 2020a). In general, the active ingredients in plants play important roles in cancer treatment. It has been reported that 20% of the active ingredients in plants, including terpenoids, flavonoids, and steroids, have significant potential as drugs (Yu et al., 2022). Interestingly, on the basis of molecular expression profiles and genomic analysis, the applications of natural plant products, especially traditional Chinese medicines, are being continuously optimized and modernized, and an increasing number of active ingredients are being discovered and utilized for cancer treatment (Hui et al., 2024).

Programmed cell death (PCD) is a cell death process regulated by a series of molecular programs. PCD currently includes apoptosis, autophagy, necroptosis, pyroptosis, ferroptosis, and cuproptosis (Mishra et al., 2018). Caspase and Bcl family proteins are central regulatory molecules in the apoptosis pathway and are closely associated with proapoptotic signals (Peng et al., 2022). ULK1, Beclin-1, LC3, and p62 are key regulatory factors in autophagy, and studies have shown that autophagy is modulated by the PI3K/Akt/mTOR, MAPK/mTOR and AMPK/mTOR pathways (Yoshida, 2017). Ferroptosis, a newly discovered PCD pathway, is closely associated with glutathione peroxidase (GPX family) and the cystine/glutamate transporter (SLC7A11). In pyroptosis, gasdermin (GSDM) family members are key mediators that are regulated by caspase-related proteins and ultimately cause cell death (Tong et al., 2022).

The use of natural plant compounds to target key signaling pathways or genes involved in PCD provides a new approach for cancer treatment. Active ingredients in plants, such as saponins, quinones, alkaloids and flavonoids, can target apoptosis or necroptosis pathways in cancer cells (Yang et al., 2023; Gali-Muhtasib et al., 2015). Terpenoids and flavonoids promote apoptosis and have been applied in the systemic treatment of patients with colon cancer, breast cancer, and liver cancer (Hui et al., 2024).

**FIGURE 1:**
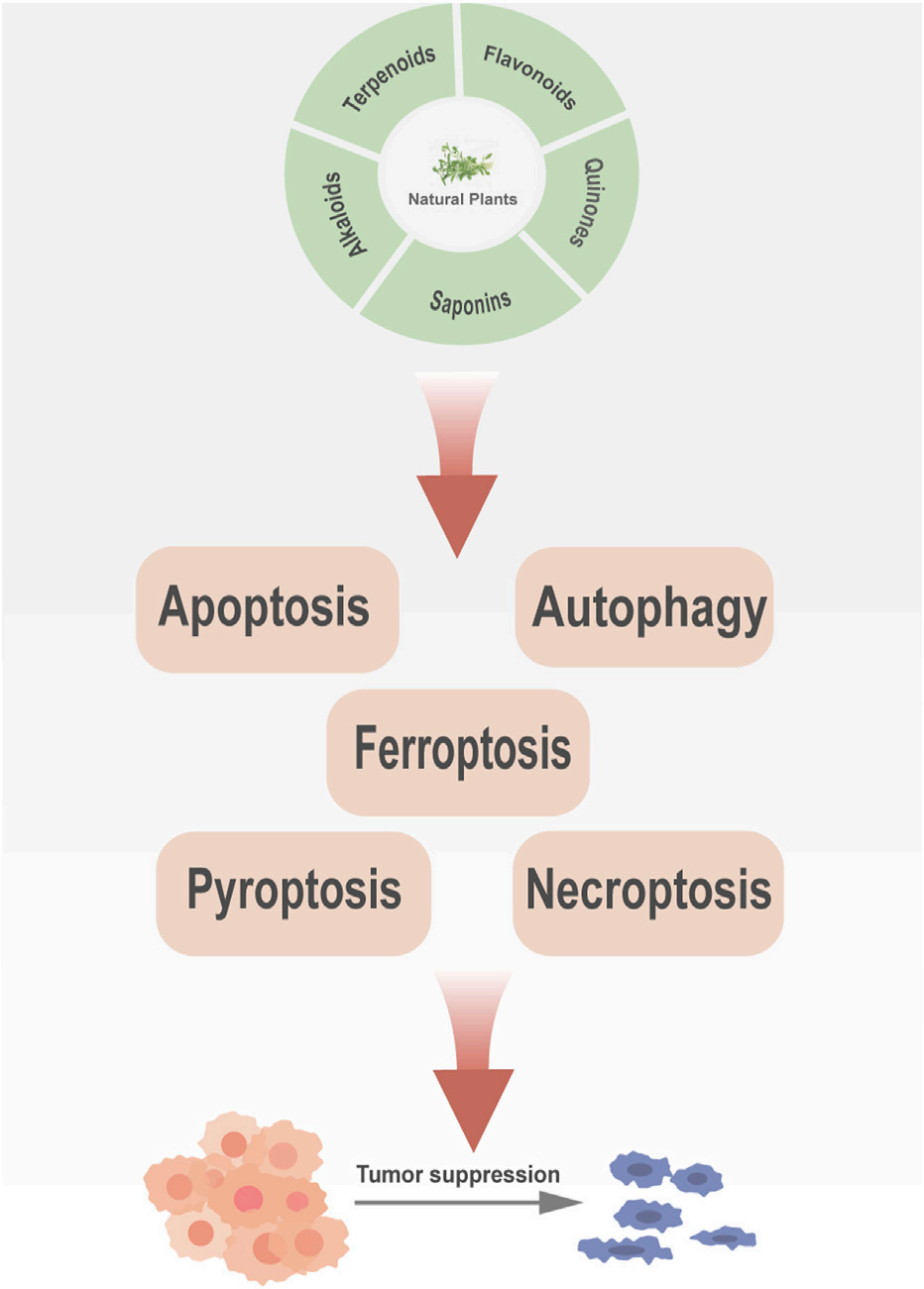
Natural plant products can inhibit cancer development via targeting different programmed cell death pathways.

This review summarizes the roles and mechanisms of major categories of natural plant active ingredients in different PCD processes and provides a strong theoretical basis for clinical cancer management (Figure 1). The types and sources of natural plant products, mechanisms involved in PCD, and cancer types described in this review are presented in Table 1, and the chemical structural formulas of the related natural plant products are presented in Table 2.

**TABLE 1:** Overview of natural plant compounds that target programmed cell death in cancer cells.

	Species	Compound	Source	Mechanism	Tumor type
**Apoptosis**	Flavonoids	Baicalin	Scutellaria baicalensis Georgi	PI3K/AKT (Sui et al., 2020b) JNK (Huang et al., 2019) AMPK/m TOR (Jia et al., 2022a)	cholangiocarcinoma (Jia et al., 2022a), lung cancer (Sui et al., 2020b), pancreatic cancer (Huang et al., 2019)
		Luteolin	Dragonhead, wild chrysanthemum flower, honeysuckle	PI3K/AKT (Wu et al., 2020a) p53 (Yoo et al., 2022) TRAIL/JNK (Feng et al., 2018; Nazim and Park, 2019a) Wnt/β-catenin (Pandurangan et al., 2013; Qin et al., 2022; Lin et al., 2017; Yang et al., 2024)	Hepatocellular carcinoma (Feng et al., 2018; Nazim and Park, 2019a), colon cancer (Yoo et al., 2022), breast cancer (Wu et al., 2020a), TNBC (Pandurangan et al., 2013), osteosarcoma (Qin et al., 2022; Lin et al., 2017), cholangiocarcinoma (Yang et al., 2024)
		Icariin	Epimedium	NF-кB (Song et al., 2020) JNK/c-JUN (Gao et al., 2023)	TNBC (Song et al., 2020), TNCB (Gao et al., 2023)
		Apigenin	*Apium graveolens* L. var. dulce DC	PI3K/AKT (Yang et al., 2018) DR5/TRAIL (Kang et al., 2018) P53 (Yang et al., 2021b; Hamadou et al., 2021)	Hepatocellular carcinoma (Yang et al., 2018; Kang et al., 2018; Şirin et al., 2020), colorectal cancer (Yang et al., 2021b; Hamadou et al., 2021)
	Terpenoids	Atractylon	Atractylodes	PI3K/AKT (Mao et al., 2022) SIRT3/p53 (Sun et al., 2022)	colorectal cancer (Mao et al., 2022), Hepatocellular carcinoma (Cheng et al., 2019), glioma (Sun et al., 2022)
		Paclitaxel	Chinese yew	PI3K/AKT/mTOR (Li et al., 2020; Li et al., 2023a) TAK1/TAB1/JNK (Yu-Wei et al., 2020; Zhang et al., 2022a) AMPK/mTOR (Rocha et al., 2011)	thyroid cancer (Yu-Wei et al., 2020) esophageal cancer (Li et al., 2023a), breast cancer (Li et al., 2020; Rocha et al., 2011),lung cancer (Rocha et al., 2011) prostate cancer (Zhang et al., 2022a)
		β-Elemene	Curcuma wenyujin	PI3K/AKT (Wang et al., 2021a) JAK2/STAT3 (Xiaomeng et al., 2020; Cai et al., 2021a) AMPK/MAPK (Wang et al., 2021a; Wang et al., 2022; Liu et al., 2020c) p53 (Yang et al., 2021a; Dong et al., 2024) PERK/IRE1α/ATF6 (Liu et al., 2017) IGF1/IGF1R (Xie et al., 2022) PUMA (Hu and Gao, 2018)	bladder cancer (Liu et al., 2020c), burkitt lymphoma (Hu and Gao, 2018), NSCLC (Liu et al., 2017), glioma (Yang et al., 2021a; Xiaomeng et al., 2020; Cai et al., 2021a), colorectal cancer (Wang et al., 2021a; Wang et al., 2022), prostate cancer (Dong et al., 2024), TNBC (Xie et al., 2022)
**Necroptosis**	Flavonoids	Apigenin	*Apium graveolens* L. var. dulce DC	RIPK1/RIPK3/MLKL (Lee et al., 2020a)	malignant mesothelioma (Lee et al., 2020a)
		Acacetin	Agastache rugosus	RIPK1/RIPK3 (Kandhari et al., 2023)	breast cancer (Kandhari et al., 2023)
		Isobavachalcone	Isobavachalcone	RIPK1/RIPK3/MLKL (Wu et al., 2022a)	TNBC (Wu et al., 2022a)
		Gambogic Acid	Gambogic	RIPK1/RIPK3/MLKL (Chen et al., 2024)	gastric cancer (Chen et al., 2024)
		Quercetin	Radix bupleuri, mulberry leaf, fructus sophorae, inula flower, hawthorn, etc	cIAP1/2/RIPK1/RIPK3/MLKL (Lomphithak et al., 2023)	cholangiocarcinoma (Lomphithak et al., 2023)
		Kaempferol	Rhizoma Kaempferiae	cIAP1/2/RIPK1/RIPK3/MLKL (Lomphithak et al., 2023)	cholangiocarcinoma (Lomphithak et al., 2023)
		Fisetin	Toxicodendron sylvestre	ZBP1/RIP3/MLKL (Liu et al., 2022)	ovarian cancer (Liu et al., 2022)
	Quinones	Shikonin	Lithospermum	RIPK1/MLKL (Chen et al., 2017; Liu et al., 2019)	pancreatic cancer (Chen et al., 2017), nasopharyngeal carcinoma (Liu et al., 2019)
		Emodin	rheum officinale	TNF-α/RIP1/RIP3 (Zhou et al., 2020) HSP90/MLKL/PGAM5 (Zhou et al., 2023)	Glioma (Zhou et al., 2020), prostate cancer (Zhou et al., 2023)
	Terpenes	Paclitaxel	Chinese yew	RIPK1/RIPK3/MLKL (Diao et al., 2016)	lung adenocarcinoma (Diao et al., 2016)
		Oridonin	Rabdosia rubescens	RIPK1/RIPK3/MLKL (Zheng et al., 2018)	renal cell carcinoma (Zheng et al., 2018)
		IDOAMP	Rosin	RIPK1/RIPK3/MLKL (Xu et al., 2022a)	prostate cancer (Xu et al., 2022a)
	Others	Matrine	radix sophorae flavescentis	RIP3/MLKL (Xu et al., 2017)	cholangiocarcinoma (Xu et al., 2017)
		tanshinol A	Salvia miltiorrhiza	RIP3/MLKL/ROS (Liu et al., 2020d)	Lung cancer (Liu et al., 2020d)
		Gallic Acid	Palm leaf rhubarb, eucalyptus robusta, dogwood, etc	MLKL (Tang and Cheung, 2019)	cervical cancer (Tang and Cheung, 2019)
**Pyroptosis**	Flavonoids	Icariin	Epimedium	NLRP3 (Zhang et al., 2022b)	gastric cancer (Zhang et al., 2022b)
		Kaempferol	Rhizoma Kaempferiae	NF-κB (Qi et al., 2024)	gastric cancer (Qi et al., 2024)
		Luteolin	Dragonhead, wild chrysanthemum flower, honeysuckle	Caspase1/GSDMD/IL-1β (Chen et al., 2022a)	colorectal cancer (Chen et al., 2022a)
		Baicalin	Scutellaria baicalensis Georgi	NLRP3/GSDMD/GSDME (Lu et al., 2023) NF-κB (Liu et al., 2024a)	gastric cancer (Liu et al., 2024a) large B-cell lymphoma (Lu et al., 2023)
		Quercetin	Bupleurum, mulberry leaves, acacia horn, Helicoverna, hawthorn, etc	NLRP3/GSDMD/GSDME (Rong et al., 2023)	gastric cancer (Rong et al., 2023)
		Galangin	Alpinia officinarum	NLRP3 (Kong et al., 2019)	glioblastoma (Kong et al., 2019)
		Nobiletin	Citrus reticulata Blanco	GSDMD/GSDME (Zhang et al., 2020b)	ovarian cancer (Zhang et al., 2020b)
		Alpinumisoflavone	Uraria crinite	NLRP3 (Zhang et al., 2020c)	Hepatocellular carcinoma (Zhang et al., 2020c)
		Isobavachalcone	Psoraleae Fructus	ERα/NLRP3 (Wu et al., 2022b)	glioblastoma (Wu et al., 2022b)
	Alkaloids	Ajmalicine	Rauvolfia Serpentina	caspase-3/GSDME (Sun et al., 2024)	Hepatocellular carcinoma (Sun et al., 2024)
		Chaetoglobosin E	Chaetomium madrasense 375	PLK1/GSDME (Chen et al., 2022b)	esophageal squamous carcinoma (Chen et al., 2022b)
		Sophflarine A	Sophora flavescens	NLRP3/caspase-1/GSDMD (Luo et al., 2023)	NSCLC (Luo et al., 2023)
	Terpenes	Mallotucin D	Croton crassifolius	NLRP3/GSDMD (Dai et al., 2022)	Hepatocellular carcinoma (Dai et al., 2022)
		Paclitaxel	Chinese yew	caspase-3/GSDME (Zhang et al., 2019)	Lung cancer (Zhang et al., 2019)
		Triptolide	thunder god vine	HK2/caspase-3/GSDME (Cai et al., 2021b; Wang et al., 2023)	Head and neck cancer (Cai et al., 2021b), melanoma (Wang et al., 2023)
		Cucurbitacin B	Cucurbitaceae plant	TLR4/NLRP3/GSDMD (Yuan et al., 2021)	NSCLC (Yuan et al., 2021)
		Tanshinone IIA	Salvia miltiorrhiza	foxp3/caspase-1/GSDMD (Wang et al., 2021b) miR145/GSDMD (Tong et al., 2020)	Nasopharyngeal carcinoma (Wang et al., 2021b) , cervical cancer (Tong et al., 2020)
		Tanshinone I	Salvia miltiorrhiza	NF-κB/caspase-3 (8)/GSDME (Wang et al., 2024a)	gastric cancer (Wang et al., 2024a)
**Ferroptosis**	Terpenes	β-Elemene	Curcuma wenyujin	TFEB/GPX4 (Zhao et al., 2023) lncRNA H19/EGFR (Xu et al., 2023) ROS/GSH/Fe2^+^ (Chen et al., 2020a)	NSCLC (Zhao et al., 2023; Xu et al., 2023), colorectal cancer (Chen et al., 2020a)
		Tanshinone IIA	Salvia miltiorrhiza	p53/SLC7A11 (Guan et al., 2020) KDM1A/PIAS4/SLC7A11 (Luo et al., 2024)	gastric cancer (Guan et al., 2020), breast cancer (Luo et al., 2024)
		Tanshinone I	Salvia miltiorrhiza	KDM4D/p53/SLC7A11 (Xia et al., 2023)	gastric cancer (Xia et al., 2023)
	Alkaloids	Sanguinarine	Celandine, purple pansy, fall back, the blood aquatic plants	ROS/BACH1/HMOX1 (Liu et al., 2024b) H2O2/ROS (Alakkal et al., 2022) STIP1/STUB/GPX4 (Xu et al., 2022b)	NSCLC (Xu et al., 2022b), cervical cancer (Alakkal et al., 2022), prostate cancer (Liu et al., 2024b)
		Camptothecin	camptotheca acuminata	Nrf2/ROS/GPX4 (Zhou et al., 2024)	Hepatocellular carcinoma (Zhou et al., 2024)
		Evodiamine	fructus evodiae	GPX4 (Yu et al., 2023)	prostate Cancer (Yu et al., 2023)
		Sinomenine	Sinomenium acutum	NCOA4/FTH1/Fe^2+^ (Zhu et al., 2024)	colorectal cancer (Zhu et al., 2024)
		Solanine	Potato, tomato, eggplant, etc	ALOX12B/ADCY4/ROS (Ma et al., 2024)	colorectal cancer (Ma et al., 2024)
		Matrine	radix sophorae flavescentis	piezo1/ROS (Jin et al., 2024)	cervical cancer (Jin et al., 2024)
		Peiminine	Any of various plants of the genus Fritillaria	Nrf2/HO-1 (Yi et al., 2024)	breast cancer (Yi et al., 2024)
		Chelerythrine	Celandine plant extracts	Nrf2/ROS/GPX4 (Elkateb et al., 2023)	ovarian cancer (Elkateb et al., 2023)
	Flavonoids	Luteolin	Whole leaf green orchid, wild chrysanthemum, honeysuckle	HO-1/LIP (Han et al., 2022) TFEB/FTH (Fu et al., 2024) HIC1/GPX4 (Zheng et al., 2023)	colorectal cancer (Zheng et al., 2023), prostate cancer (Fu et al., 2024), renal cell carcinoma (Han et al., 2022)
		Baicalin	Scutellaria baicalensis Georgi	p53/SLC7A11 (Yuan et al., 2023; Shao et al., 2024) FTH1 (Wen et al., 2024; Kong et al., 2021) Nrf2/HO-1/GPX4 (Wen et al., 2023b)	gastric cancer (Yuan et al., 2023; Shao et al., 2024),bladder cancer (Kong et al., 2021),oral squamous cell carcinoma (Wen et al., 2024),osteosarcoma (Wen et al., 2023b)
		Quercetin	Bupleurum, mulberry leaves, acacia horn, Helicoverna, hawthorn, etc	TFEB/FTH (Wang et al., 2021c; An and Hu, 2022)	Breast cancer (An and Hu, 2022), colorectal cancer (Wang et al., 2021c), Hepatocellular carcinoma (Wang et al., 2021c)
	Others	Erianin	Dendrobium chrysotoxum	Nrf2/HO-1 (Xiang et al., 2021a) Ca^2+/^Fe^2+^ (Chen et al., 2020b)	bladder cancer (Xiang et al., 2021a),lung cancer (Chen et al., 2020b)
		Ginkgetin	Gingko	Nrf2/HO-1 (Lou et al., 2021)	NSCLC (Lou et al., 2021)
**Autophagy**	Saponins	Tubeimoside-1	Bolbostemma paniculatum Franquet	PI3K/AKT/mTOR (Jiang et al., 2019)	breast cancer (Jiang et al., 2019)
		Sasanquasaponin Ⅲ	tea plant	PI3K/AKT/mTOR (Liang et al., 2019)	melanoma (Liang et al., 2019)
		Polyphyllin Ⅵ	Paris polyphylla	PI3K/AKT/mTOR (Teng et al., 2019)	NSCLC (Teng et al., 2019)
		Platycodin D (PD)	Platycodon grandiflorum	MAPK/mTOR (Han et al., 2024)	colorectal cancer (Han et al., 2024)
		paris saponin Ⅶ	Trillium	AMPK/mTOR (Xiang et al., 2021b) MST2-MOB1-LATS1/YAP/Hippo (Xiang et al., 2022)	NSCLC (Xiang et al., 2021b), breast cancer (Xiang et al., 2022)
		Ginsenoside Rk3	Panax ginseng	Akt/mTOR (Qu et al., 2023)	hepatocellular carcinoma (Qu et al., 2023)
		Ginsenoside Rg5	Ginseng	MAPK/mTOR (Liu and Fan, 2019)	gastric cancer (Liu and Fan, 2019)
		Ginsenoside CK	ginseng	Beclin-1/Atg5/LC3-II/P62 (Yin et al., 2021)	cervical cancer (Yin et al., 2021)
		Aescin	Heavenly Master Millet	Akt/mTOR (Singh et al., 2023)	lung cancer (Singh et al., 2023)
	Terpenes	β-Elemene	Curcuma wenyujin	LC3Ⅰ/LC3Ⅱ (Guan et al., 2014; Zhang et al., 2023b) PI3K/AKT/mTOR (Zhan et al., 2012a; Liu et al., 2011) MAPK/Erk/mTOR (Zhan et al., 2012a) AMPK/mTOR (Wang et al., 2022) EGFR/AKT (Mu et al., 2016; Zhang et al., 2020d) PFKFB4 (Jalal et al., 2022) ATG5/ATG7 (Liu et al., 2020e)	NSCLC (Liu et al., 2012a; Liu et al., 2020e), melanoma (Jalal et al., 2022), glioblastoma (Mu et al., 2016), colorectal cancer (Wang et al., 2022; Zhang et al., 2020d), breast cancer (Guan et al., 2014), renal cell carcinoma (Zhan et al., 2012a), gastric cancer (Liu et al., 2011), Ewing’s sarcoma (Zhang et al., 2023b)
	Flavonoids	luteolin	Whole leaf green orchid, wild chrysanthemum, honeysuckle	SGK1/FOXO3/LC3Ⅱ (Wu et al., 2023) TRAIL (Nazim and Park, 2019a)	hepatocellular carcinoma (Lee and Kwon, 2019; Nazim and Park, 2019a), glioblastoma (Chakrabarti and Ray, 2016; Lee et al., 2021a), TNBC (Wu et al., 2023), ovarian cancer (Liu et al., 2018)
		Baicalin	Scutellaria baicalensis Georgi	PI3K/AKT/mTOR (Pang et al., 2022a; Lin et al., 2013a; Zhang et al., 2012a; Gou et al., 2009a) ERK1/2 (Pang et al., 2022a) β-catenin (Pang et al., 2022a) LC3Ⅱ/Beclin1 (Wang et al., 2020)	bladder cancer (Lin et al., 2013a),nasopharyngeal cancer (Wang et al., 2020), hepatocellular carcinoma (Zhang et al., 2012a; Gou et al., 2009a), osteosarcoma (Pang et al., 2022a)

**TABLE 2:** Structural formula of natural plant active ingredients that target programmed cell death in cancer cell.

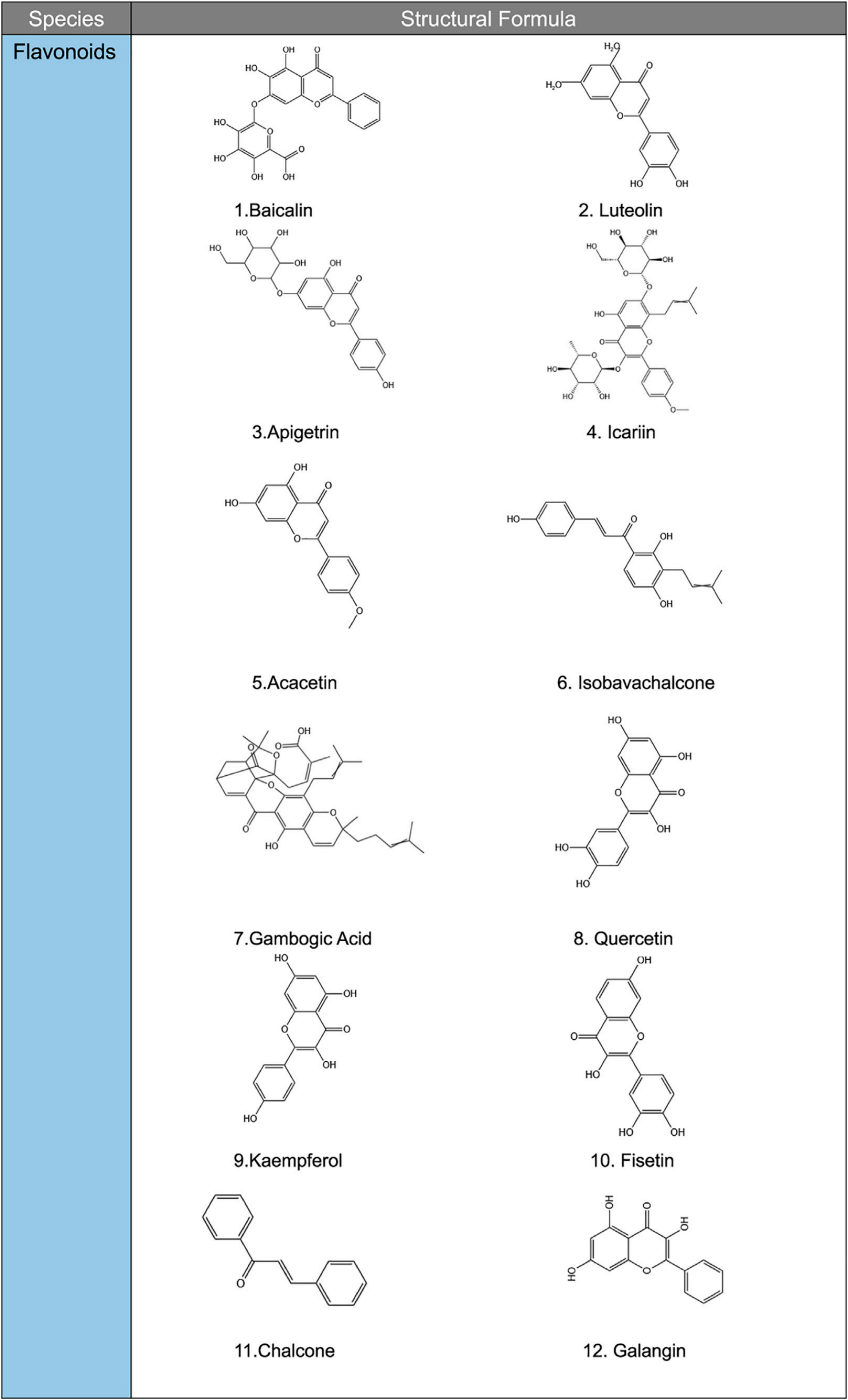
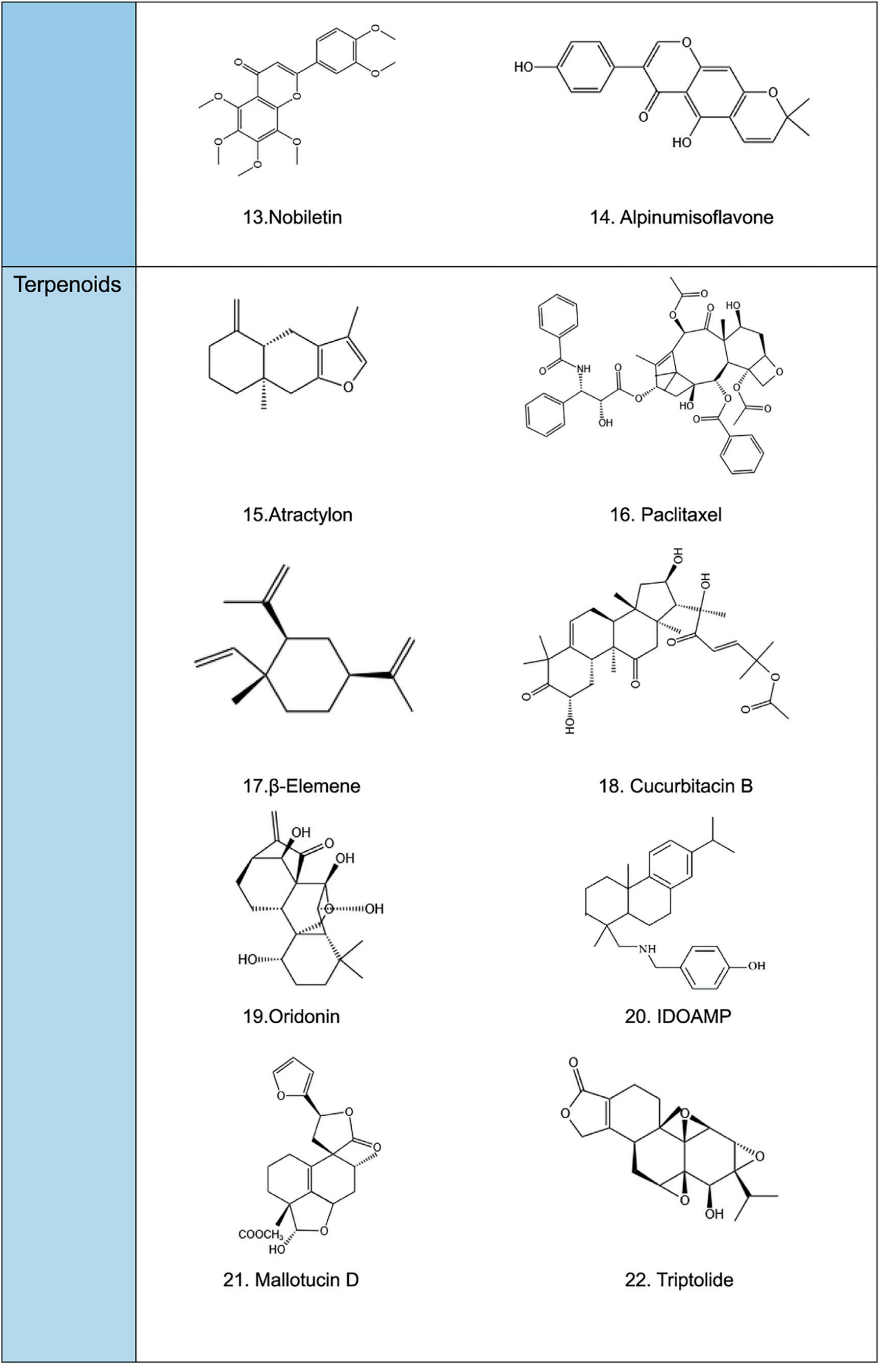
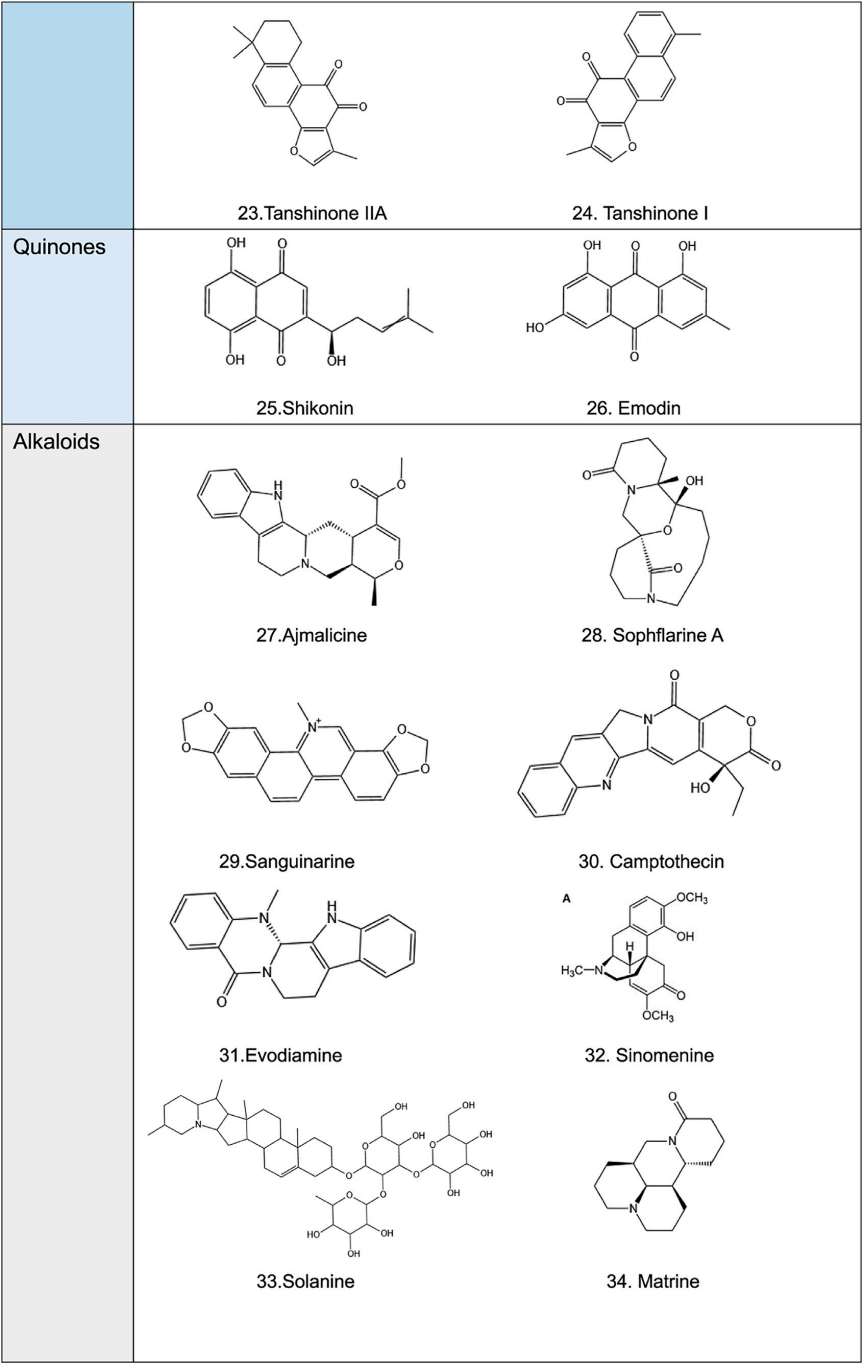
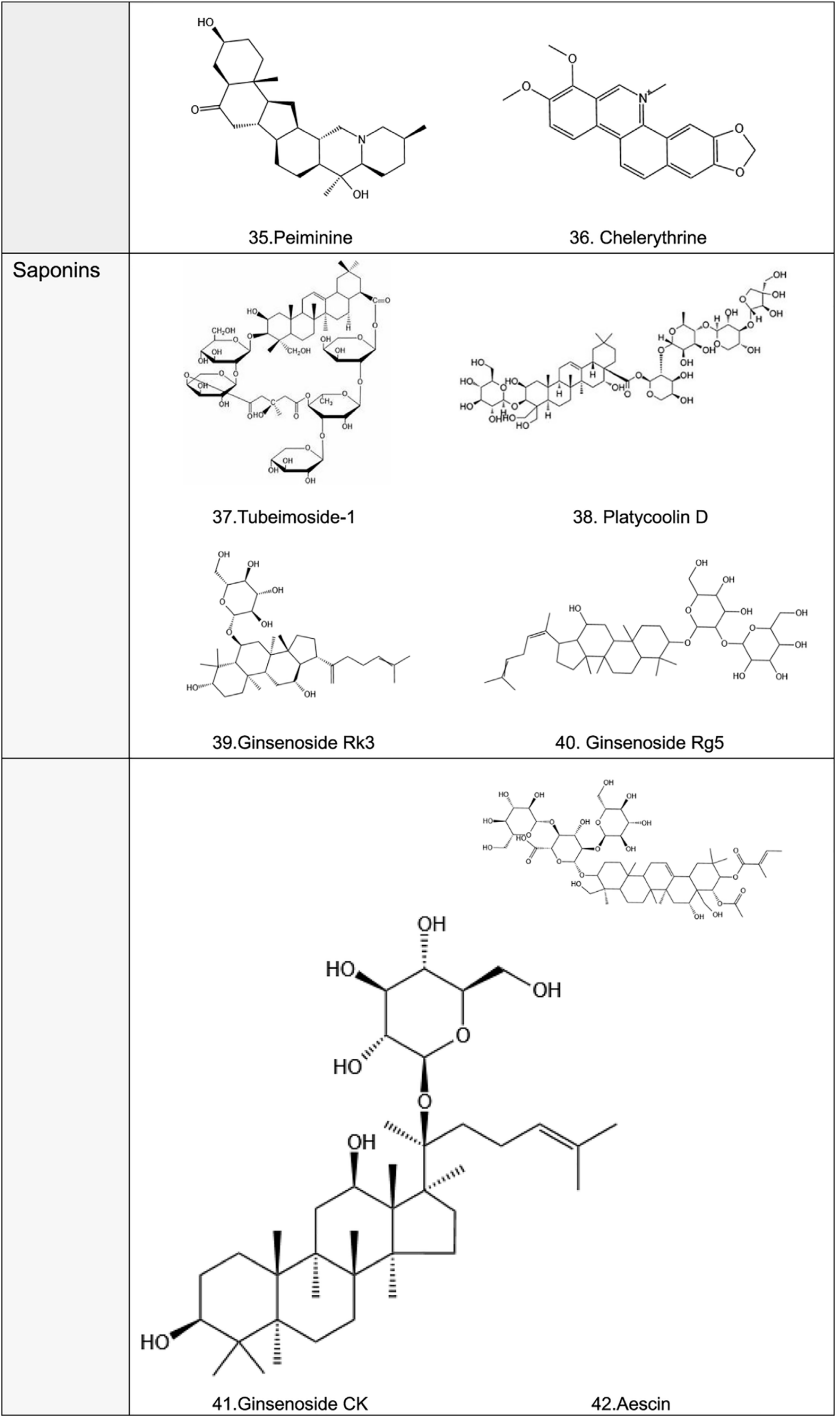

## 2 Modulation of PCD in cancer via natural plant compounds

### 2.1 Apoptosis

**FIGURE 2:**
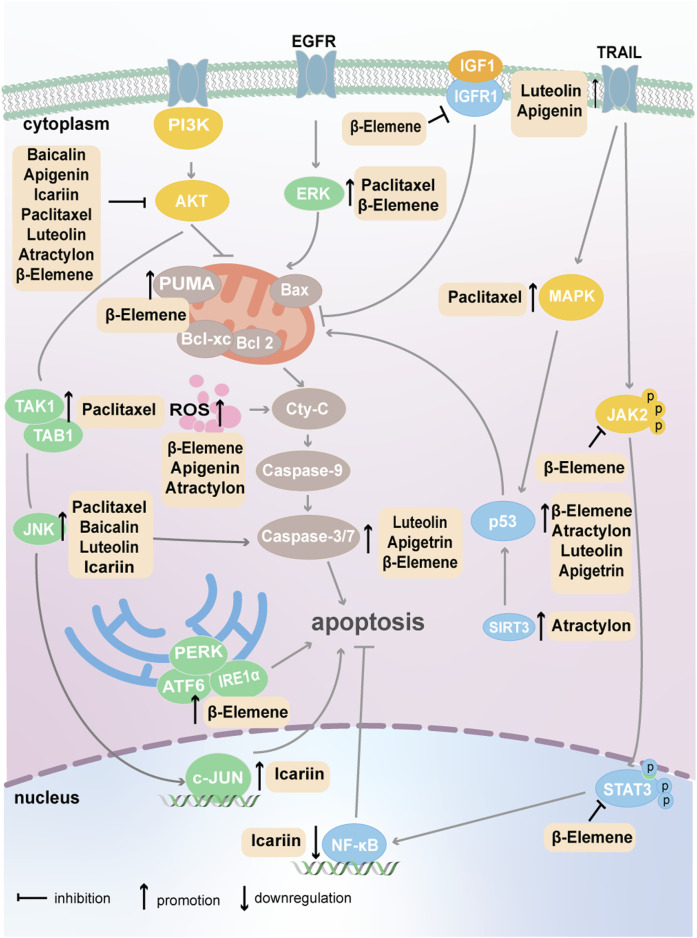
Plant natural compounds induce the apoptosis of cancer cells via mitochondrial and endoplasmic reticulum pathways.

Apoptosis was the first form of PCD to be identified; it is a strictly controlled mode of cell death characterized by cell shrinkage, nuclear condensation, and DNA fragmentation (Mishra et al., 2018). The natural plant compounds that target tumor cell apoptosis are summarized in Figure 2.

#### 2.1.1 Terpenoids

Terpenoids are a large class of organic compounds widely distributed in plants, insects, microorganisms and marine organisms. Some natural terpenoids have attracted increasing attention from researchers because of their strong anticancer and anti-inflammatory activities.

##### 2.1.1.1 β-Elemene

β-Elemene comes from the root of Curcuma wenyujin and is now widely used to treat lung cancer, liver cancer, respiratory and digestive tract tumors, and malignant pleural effusion (Liu et al., 2020a). Trending with the latest innovative concept of the “molecular compatibility theory”, the research and development of β-Elemene dosage forms has become more diverse (Jiang et al., 2024). In addition to widely used β-Elemene emulsions, PEGylated β-Elemene liposomes exhibit enhanced antitumor effects (Zhai et al., 2020). Moreover, structural modification products of β-Elemene, nitric oxide derivatives (Zhu et al., 2023; Bai et al., 2022), hydroxyl carboximates (Gao et al., 2022), and macrocycles (Qi et al., 2022) all have significant antitumor effects.

Among the antitumor mechanisms of β-Elemene, the promotion of cancer cell apoptosis is one of the main mechanisms and involves the regulation of multiple signaling pathways and proteins, such as MAPK, PI3K/AKT, AMPK, STAT3 and p53 (Bai et al., 2021). β-Elemene can trigger apoptosis by activating the intrinsic pathway [mediated by a reduction in the mitochondrial membrane potential and release of cytochrome c from mitochondria or excessive endoplasmic reticulum (ER) stress] or extrinsic pathway (mediated by death receptors) and ultimately upregulate caspase-3 expression to initiate apoptosis (Liu et al., 2020b).

The combination of β-Elemene with arsenene nanodots acts on the mitochondrial apoptosis pathway, drives the production of excess reactive oxygen species (ROS), results in cytochrome c release, and induces cancer cell apoptosis (Liu et al., 2020b; Liu et al., 2021). β-Elemene alone also affects the generation of ROS in cancer; it can reduce the GSH content in lung cancer cells and inhibit glutathione synthesis, leading to the massive production of ROS (Song et al., 2023). In addition to effects on the intrinsic mitochondrial pathway, the effect of β-Elemene on cell apoptosis can also occur through the ER-mediated apoptosis pathway. β-Elemene can upregulate the expression of the unfolded protein response-related proteins PERK, IRE1α and ATF6, increase ER stress, and ultimately promote non-small cell lung cancer (NSCLC) cell apoptosis (Liu et al., 2017).

Additionally, β-Elemene can activate the AMPK pathway and inhibit the MAPK/ERK and AKT/mTOR pathways, resulting in the apoptosis of lung cancer cells (Wang J. et al., 2021). In bladder cancer and colorectal cancer (CRC), β-Elemene can induce apoptosis by generating a large amount of ROS and activating the AMPK pathway (Wang et al., 2022; Liu et al., 2020c). Yang et al. discovered that high concentrations of β-Elemene significantly stimulate the expression of p53 and ultimately cause glioma cells to undergo apoptosis (Yang D. et al., 2021). Intriguingly, another study showed that β-Elemene significantly promotes apoptosis in prostate cancer cells, an effect that may be associated with the p53 pathway (Dong et al., 2024). In ovarian cancer, β-Elemene significantly suppresses STAT3 phosphorylation, thereby promoting cell apoptosis (Xiaomeng et al., 2020). Cai et al. revealed that β-Elemene inhibits Janus-activated kinase 2 (JAK2) and Src phosphorylation and inactivated the downstream STAT3 pathway, leading to the massive production of ROS and the apoptosis of glioma cells (Cai SZ. et al., 2021).

In other mechanistic studies, β-Elemene was shown to suppress the growth of triple-negative breast cancer (TNBC) cells by reducing the expression of insulin-like growth factor 1 (IGF1), insulin-like growth factor 1 receptor (IGF1R) and Bcl-2; promoting the expression of cleaved caspase-3; and thus initiating apoptosis (Xie et al., 2022). In Burkitt’s lymphoma, β-Elemene stimulates the expression of p53 upregulated modulator of apoptosis (PUMA), a proapoptotic gene, which subsequently increases the expression of proteins such as Bax and Bak to induce cell apoptosis (Hu and Gao, 2018).

##### 2.1.1.2 Atractylon

Atractylon is derived from the perennial herb *Atractylodes macrocephala* of the Asteraceae family. It has antitumor, antibacterial, anti-inflammatory, antiaging, antioxidant, and neuroprotective activities (Cheng et al., 2016).

Atractylon suppresses the PI3K/AKT/mTOR signaling pathway and downregulates the expression of PI3K, AKT, mTOR and Bcl-2 to induce the apoptosis of CRC cells (Mao et al., 2022). In glioma, atractylon can promote apoptosis by increasing the expression of sirtuin 3 and downstream p53 and Bcl-2 (Sun et al., 2022). Although molecular docking revealed that atractylon can bind to sirtuin 3, the detailed mechanism by which atractylon controls the expression of sirtuin 3 remains unclear. Cheng et al. reported that the apoptosis of liver cancer cells increases after treatment with atractylon, which was shown to increase ROS levels and Bax and cleaved caspase-3 expression and inhibit Bcl-2 expression (Cheng et al., 2019).

##### 2.1.1.3 Paclitaxel

Paclitaxel is derived from the yew tree. Owing to its highly efficient biological activity, unique mechanism of action, and broad antitumor spectrum, paclitaxel has been widely used in clinical cancer treatment (Zhao et al., 2022). The main mechanism of action of paclitaxel is binding to tubulin, especially β-tubulin, which inhibits microtubule depolymerization and blocks spindle formation and cell division. Paclitaxel blocks the mitotic process, causing tumor cells to stay in G2 and M phases, which triggers apoptotic signals and ultimately leads to the death of tumor cells (Hardin et al., 2017). The regulatory mechanism by which paclitaxel induces cancer cell apoptosis has also been well studied, and paclitaxel-induced apoptosis is known to be related to the PI3K/AKT, AMPK, mTOR and JNK pathways. For example, in breast cancer, paclitaxel inhibits the PI3K/AKT signaling pathway, upregulates cleaved caspase-3 and Bax expression, and downregulates Bcl-2 expression, and the combination of the AKT inhibitor Capivasertib with paclitaxel significantly prolongs the progression-free survival and overall survival of breast cancer patients (Li et al., 2020). Furthermore, studies have shown that paclitaxel can increase the protein levels of transforming growth factor‐beta‐activated kinase 1 (TAK1) and TAK1‐binding protein 1 (TAB1), which are modulators of the JNK pathway, and increase the phosphorylation of JNK and the expression of cleaved caspase-7 and PARP, ultimately leading to the apoptosis of thyroid cancer cells (Yu-Wei et al., 2020). In prostate cancer, HIF-1α expression has been shown to significantly increase after paclitaxel treatment, stimulating the activation of JNK/caspase-3 signaling and promoting cell apoptosis (Zhang Y. et al., 2022).

The mTOR signaling pathway is involved in many cancers, and paclitaxel may have dual effects on this pathway. Paclitaxel can promote mucin20 expression and subsequently activate the mTOR pathway, thereby inducing the apoptosis of esophageal cancer cells (Li M. et al., 2023). However, the relationship between mucin20 and the mTOR pathway needs to be elucidated. Nevertheless, in breast cancer and lung cancer cells, paclitaxel activates AMPK, inhibits mTOR signaling and increases apoptosis (Rocha et al., 2011). This discrepancy in results means that how paclitaxel modulates the mTOR pathway needs to be discussed case by case according to different cancer types.

#### 2.1.2 Flavonoids

##### 2.1.2.1 Baicalin

Baicalin is a flavonoid compound extracted from the root of *Scutellaria baicalensis* Georgi. In addition to its antibacterial, diuretic, and anti-inflammatory effects, baicalin is currently also widely used to treat various cancers (Wen Y. et al., 2023). Baicalin induces the apoptosis of cholangiocarcinoma cells by activating the AMPK pathway and inhibiting the mTOR pathway (Jia et al., 2022a). Similarly, a study on lung cancer showed that baicalin can inactivate the AKT/mTOR pathway and promote apoptosis (Sui et al., 2020b). In pancreatic cancer, baicalin triggers cell apoptosis by targeting the JNK pathway (Huang et al., 2019).

##### 2.1.2.2 Luteolin

Luteolin is a natural flavonoid compound found in many plants. Its anti-cancer mechanisms involve reversing the epithelial-mesenchymal transition (EMT), blocking the cell cycle, increasing ROS and regulating tumor cell apoptosis by controlling multiple cell signaling pathways (Imran et al., 2019). In tamoxifen-resistant breast cancer, luteolin promotes cell apoptosis by suppressing the PI3K/AKT/mTOR pathway. Further mechanistic research has revealed that luteolin stimulates the expression of mixed-lineage leukemia 3 (MLL3) and that MLL3 epigenetically inhibits the expression of Ras family genes, which are activators of PI3K (Wu HT. et al., 2020). Moreover, luteolin can function as a sensitizer in liver cancer when combined with sorafenib or tumor necrosis factor-related apoptosis-inducing ligand (TRAIL), a cytokine that initiates cancer cell apoptosis, synergistically causing cell apoptosis by activating the JNK signaling pathway (Feng et al., 2018; Nazim and Park, 2019a). In addition to affecting signaling pathways, luteolin significantly increases p53 phosphorylation and colon cancer cell apoptosis (Yoo et al., 2022).

In addition, another unique anti-cancer mechanism of luteolin is inhibiting the Wnt/β-catenin signaling pathway (Pandurangan et al., 2013). Luteolin has been shown to reverse the EMT by down-regulating β-catenin in TNBC, and to attenuate chemoresistance in osteosarcoma by inhibiting the β-catenin signaling pathway (Qin et al., 2022; Lin et al., 2017). Recently, Yang et al.‘s molecular docking analysis revealed that luteolin could form a stable hydrogen bond with β-catenin, leading to the loss of its α-helix structure and inducing its conformational change, ultimately inhibiting Wnt signaling and promoting the apoptosis of cholangiocarcinoma (CCA) cells (Yang et al., 2024).

##### 2.1.2.3 Icariin

Epimedium is a perennial herb in the Berberidaceae family, and icariin is its main active ingredient. Icariin is a natural flavonoid monomer with various functions in the biological processes of cancer (Zhang X. et al., 2023). Song et al. reported that icariin suppresses the protein level of the NF‐κB p65 subunit in the nucleus; stimulates the expression of sirtuin 6, which deacetylates histone H3 lysine 9 around the promoters of NF‐κB target genes; and impairs NF‐κB signaling to induce the apoptosis of TNBC cells (Song et al., 2020). Additionally, in breast cancer, icariin reduces the protein expression of JNK and the phosphorylation levels of JNK and c-JUN, ultimately inducing cell apoptosis. Molecular docking has shown that icariin can bind c-Jun through hydrogen bonds, with a binding affinity of −11 kcal mol^−1^ (Gao et al., 2023).

In addition to icariin, other active ingredients in epimedium, such as baohuoside-1, also target apoptosis to exert anticancer effects. Baohuoside-1 inactivates the mTOR signaling pathway in hepatocellular carcinoma (HCC) in a dose-dependent manner, reduces Bcl-2 expression, and enhances cleaved caspase3 and Bax expression (Guo et al., 2020).

##### 2.1.2.4 Apigenin

Apigenin is a flavonoid compound that has significant preventative effects against HCC, CRC, breast cancer, gastric cancer and prostate cancer. In HCC, apigenin activates apoptosis through different mechanisms. First, it inhibits the classic signaling pathway of apoptosis, the PI3K/AKT pathway, resulting in the significant downregulation of Bcl-2 expression and significant upregulation of Bax and Bak expression (Yang et al., 2018). Second, apigenin stimulates the expression of death receptor 5 (DR5), which interacts with TRAIL and triggers canonical apoptotic signaling (Kang et al., 2018). Moreover, apigenin can enhance the anti-HCC effect of sorafenib by inducing apoptosis, but the detailed mechanism has yet to be been reported (Şirin et al., 2020).

Apigenin can decrease 5-fluorouracil (5-FU) resistance in CRC cells by targeting apoptosis; mechanistically, it promotes the expression of p53 and the production of ROS (Yang C. et al., 2021). Moreover, Hamadou et al. reported that apigenin and its derivatives induce the apoptosis of CRC cells through p53 (Hamadou et al., 2021).

### 2.2 Necroptosis

Necroptosis is a regulated form of necrosis, a process of self-destruction that is activated by preventing apoptosis. Necroptosis differs from apoptosis and other forms of PCD because it is independent of caspase activity and is primarily mediated by receptor interacting serine/threonine protein kinase 1 (RIPK1), RIPK3, and mixed lineage kinase domain-like (MLKL) (Gong et al., 2019).

#### 2.2.1 Flavonoids

##### 2.2.1.1 Apigenin, acacetin and fisetin

Research on the role of natural flavonoids in necroptosis is limited and has focused mainly on the RIPK family and MLKL. Apigenin significantly inhibits the viability of malignant mesothelioma cells by activating RIPK3 and MLKL, increasing ROS, and thereby promoting the occurrence of necroptosis (Lee YJ. et al., 2020). Similarly, in breast cancer, acacetin can promote the expression of RIPK1 and RIPK3 and the production of ROS, resulting in cell necroptosis (Kandhari et al., 2023). Liu et al. reported that fisetin increases the expression of Z-DNA-binding protein 1, a key stimulator of cell necroptosis, activates the RIPK3/MLKL pathway and induces necroptosis in ovarian cancer (Liu et al., 2022; Lomphithak et al., 2023).

##### 2.2.1.2 Quercetin, kaempferol, isobavachalcone and gambogic acid

Quercetin and kaempferol, which are natural flavonoids, when used in combination with Smac mimetics, induce the proteasomal degradation of cellular inhibitor of apoptosis proteins one and 2 (cIAP1/2) and synergistically kill cholangiocarcinoma cells through RIPK1/RIPK3/MLKL-mediated necroptosis (Lomphithak et al., 2023). The treatment of TNBC cells with isobavachalcone downregulates AKT and phosphorylated AKT (p-AKT) expression and upregulates RIPK3, p-RIPK3 and MLKL expression, subsequently inducing necroptosis (Wu CZ. et al., 2022). Gambogic acid derived from Garcinia hanburyi trees promotes gastric cancer cell death through necroptosis; regarding the mechanism of action, gambogic acid increases the p-RIPK1/RIPK1, p-RIPK3/RIPK3, and p-MLKL/MLKL ratios; enhances necrosome complex formation; activates the downstream effector proteins phosphoglycerate mutase family member 5 (PGAM5) and Drp-1 (Chen et al., 2024).

#### 2.2.2 Quinones

##### 2.2.2.1 Shikonin

Owing to their “dominant skeletons” in medicinal chemistry, the advantages of quinones in cancer treatment are gradually becoming apparent. Shikonin is derived from the Chinese herb *Lithospermum erythrorhizon*. Studies have shown that shikonin induces necroptosis in pancreatic cancer cells. Importantly, the mechanism of action might be cell-type specific. Shikonin functions mainly through RIPK1 in the PANC-1 cell line and has no obvious effect on RIPK3; however, in AsPC-1 cells, it targets RIPK3 to induce cell necroptosis (Chen et al., 2017), and in nasopharyngeal carcinoma, shikonin upregulates both RIPK1 and RIPK3 expression and MLKL expression to promote necroptosis (Liu et al., 2019).

##### 2.2.2.2 Emodin

Emodin is an anthraquinone compound, and studies of glioma have shown that it increases the protein levels of TNF-α, RIPK1, RIPK3 and MLKL *in vivo* and *in vitro*, resulting in cell necroptosis (Zhou et al., 2020). In prostate cancer, emodin treatment causes necroptosis-mediated cell death by stimulating the expression of HSP90 and PGAM5, a central modulator of mitochondrial fission, leading to the massive generation of ROS and necroptosis (Zhou et al., 2023).

#### 2.2.3 Terpenoids

##### 2.2.3.1 Oridonin and paclitaxel

Oridonin is a diterpenoid derived from *Rabdosia rubescens*, and in renal cancer, it can upregulate the expression of RIPK1 and RIPK3, promoting necroptosis (Zheng et al., 2018).

c-Src phosphorylates caspase 8 at tyrosine 380 in lung adenocarcinoma cells, and paclitaxel triggers necroptosis through p-caspase 8. After paclitaxel treatment, p-caspase 8 interacts with RIPK1 and RIPK3, which reinforces RIPK1 and RIPK3 binding, resulting in the activation of the RIPK1/RIPK3/MLKL pathway (Diao et al., 2016).

##### 2.2.3.2 IDOAMP

The Rosin derivative IDOAMP is a diterpenoid. In prostate cancer, IDOAMP inhibits the binding of Aurora A to RIPK1 and RIPK3, and these interactions block necrosome activation. Moreover, IDOAMP stimulates the binding of RIPK1 to RIPK3 or MLKL and the phosphorylation of RIPK1, RIPK3 and MLKL, thereby inducing necroptosis in cells (Xu H. et al., 2022).

#### 2.2.4 Other natural compounds

##### 2.2.4.1 Progenin III, gallic acid, tanshinol a and matrine

Many other natural medicines from plants have clinical application value because they play roles in the mechanism of necroptosis. For example, the natural medicine progenin III derived from *Raphia vinifera* P. Beauv induces necroptosis in leukemia cells; however, the specific mechanism has not yet been elucidated (Mbaveng et al., 2020). In cervical cancer, gallic acid causes cancer cell death through the necroptosis pathway (Tang and Cheung, 2019). Interestingly, tanshinol A induces necroptosis via MLKL but not through RIPK1 or RIPK3 in lung cancer; it causes the massive production of ROS, increases the phosphorylation level of MLKL and promotes MLKL transfer to the plasma membrane to execute necroptosis (Liu X. et al., 2020). Matrine, an alkaloid derived from *Sophora flavescens*, upregulates RIPK3 expression and promotes MLKL translocation from the cytoplasm to the cell membrane, ultimately inducing necroptosis in cholangiocarcinoma (Xu et al., 2017).

### 2.3 Pyroptosis

Pyroptosis, also known as inflammatory PCD, is characterized by the formation of plasma membrane pores by gasdermin family proteins. Some studies have shown that pyroptosis is regulated by multiple signaling pathways and noncoding RNAs and can control cancer cell proliferation, invasion and metastasis (Fang et al., 2020).

#### 2.3.1 Flavonoids

##### 2.3.1.1 Baicalin and nobiletin

In diffuse large B-cell lymphoma cells, baicalin dose-dependently regulates the production of ROS and increases the expression of NACHT, LRR and PYD domains-containing protein 3 (NLRP3), gasdermin-D N terminal (GSDMD-N), and GSDME-N, leading to pyroptosis (Lu et al., 2023). A study by Liu et al. revealed that baicalin significantly promotes pyroptosis in gastric cancer through the generation of ROS and the upregulation of NLRP3 and GSDMD-N expression; further research revealed that baicalin also increases the protein level of NF-κB and activates this pathway to trigger pyroptosis (Liu J. et al., 2024). Nobiletin is a flavonoid isolated from citrus fruits, and the treatment of ovarian cancer cells with nobiletin significantly reduces the mitochondrial membrane potential and induces ROS production, promoting GSDMD/GSDME-mediated pyroptosis (Zhang et al., 2020b).

##### 2.3.1.2 Chalcones, kaempferol and luteolin

Chalcones belong to the flavonoid family and are commonly found in edible and medicinal plants. After structural modification, chalcone derivatives significantly inhibit lung cancer cell proliferation and growth through the ROS-mediated pyroptosis pathway (Zhu et al., 2018). In gastric cancer, kaempferol, like baicalin, activates the NF-κB pathway, thereby upregulating the expression of caspase-1, GSDMD, and IL-18 (Qi et al., 2024). Luteolin significantly promotes the expression of caspase 1, GSDMD and IL-1β in CRC cells, thereby inducing pyroptosis (Chen Y. et al., 2022).

##### 2.3.1.3 Quercetin and isobavachalcone

As previously mentioned, studies have shown that in cancer treatment, quercetin triggers cell apoptosis; in addition, recent reports have indicated that quercetin affects pyroptosis. For example, quercetin induces pyroptosis in gastric cancer cells by increasing the expression of pyroptosis markers such as GSDMD, GSDME, and NLRP3 in a concentration-dependent manner (Rong et al., 2023). In contrast, isobavachalcone prevents glioblastoma development by inhibiting pyroptosis and triggering apoptosis. Mechanistically, molecular docking and surface plasmon resonance assays revealed that isobavachalcone directly binds estrogen receptor α (ERα), an NLRP3 transcription factor, thus inhibiting NLRP3 transcription and the expression of caspase 1, GSDMD and IL-1β (Wu Y. et al., 2022).

##### 2.3.1.4 Alpinum isoflavone, icariin and galangin

Alpinum isoflavone, a flavonoid isolated from derriseriocarpa, inhibits HCC cell proliferation and metastasis by inducing pyroptosis through NLRP3 inflammasome activation (Zhang Y. et al., 2020). circCACNA2D1 expression is downregulated in gastric cancer; it sponges miR-223-3p, and miR-223-3p targets NLRP3. Icariin modulates this signaling axis and induces pyroptosis (Zhang F. et al., 2022). In glioblastoma, galangin suppresses cell proliferation by inducing pyroptosis through the upregulation of GSDME expression, with no effect on GSDMD expression (Kong et al., 2019).

#### 2.3.2 Alkaloids

##### 2.3.2.1 Camptothecin, matrine, chaetoglobosin E, ajmalicine and sophorin A

Alkaloids are a class of nitrogen-containing alkaline organic compounds that exist mainly in plants. Among these compounds, research has confirmed that camptothecin and matrine have antitumor effects but that their mechanisms of action are different. Chaetoglobosin E activates pyroptosis by targeting polo-like kinase one in esophageal squamous cell carcinoma, potentially suppressing GSDME activation (Chen JH. et al., 2022). Ajmalicine is a small-molecule alkaloid, and Sun et al. reported that it promotes pyroptosis in the H22 liver cancer cell line, mainly by increasing ROS production and upregulating the expression of caspase 3 and GSDME-N (Sun et al., 2024). Sophorin A, a novel matrine derivative isolated from *Sophora japonica*, promotes NSCLC cell death by inducing pyroptosis through NLRP3/caspase-1/GSDMD signaling pathway activation (Luo et al., 2023).

#### 2.3.3 Terpenoids

##### 2.3.3.1 Tanshinone I and tanshinone IIA

Tanshinones, a class of compounds extracted from the roots of Danshen, include tanshinone I, tanshinone IIA, and cryptotanshinone. In gastric cancer, tanshinone I suppresses cisplatin-resistant cell growth by activating the NF-κB/caspase-3/8/GSDME signaling pathway and stimulating pyroptosis (Wang G. et al., 2024). Treatment with tanshinone IIA decreases the expression of miR-125b, which targets the transcription factor foxp3 and suppresses caspase-1 and GSDMD-N expression, ultimately increasing pyroptosis and inhibiting the proliferation of nasopharyngeal carcinoma cells; however, the relationship between foxp3 and caspase-1/GSDMD is unclear (Wang Y. et al., 2021). In cervical cancer, tanshinone IIA stimulates the expression of miR-145, GSDMD and IL-1β to cause HeLa cell pyroptosis, but how miR-145 modulates pyroptosis-related gene expression needs to be clarified (Tong et al., 2020).

##### 2.3.3.2 Triptolide

Triptolide is extracted from the plant *Tripterygium wilfordii*, and studies have shown that it can reduce the protein level of mitochondrial hexokinase II, which blocks the translocation of BAD and BAX proteins to mitochondria and the activation of caspase 3, thus preventing the cleavage of GSDME and pyroptosis to eliminate head and neck cancer cells (Cai J. et al., 2021). On this basis, Wang et al. modified the dosage form of triptolide and applied it to melanoma; triptolide nanoparticles more efficiently enhanced caspase-3-mediated GSDME cleavage and induced cell pyroptosis (Wang et al., 2023).

##### 2.3.3.3 Mallotucin D, Paclitaxel and Cucurbitacin B.

Mallotucin D is a diterpenoid compound from *Croton crassifolius* that increases the expression of NLRP3, the GSDMD-N/GADMD ratio and mature IL-1β/pro-IL-1β ratio, thus inducing pyroptosis in HCC (Dai et al., 2022). Paclitaxel has been widely used in cancer treatment; it induces caspase 3 activation and GSDME cleavage in a time-dependent manner in lung cancer (Zhang et al., 2019). Another natural triterpenoid, cucurbitacin B, induces NSCLC cell pyroptosis by promoting the activation of the NLRP3 inflammasome. Further investigations revealed that cucurbitacin B directly binds to Toll-like receptor 4 (TLR4) and stabilizes it, while TLR4 activates the NLRP3 inflammasome. Moreover, cucurbitacin B promotes mitochondrial ROS generation and thus triggers GSDMD-dependent cell pyroptosis (Yuan et al., 2021).

### 2.4 Ferroptosis

**FIGURE 3:**
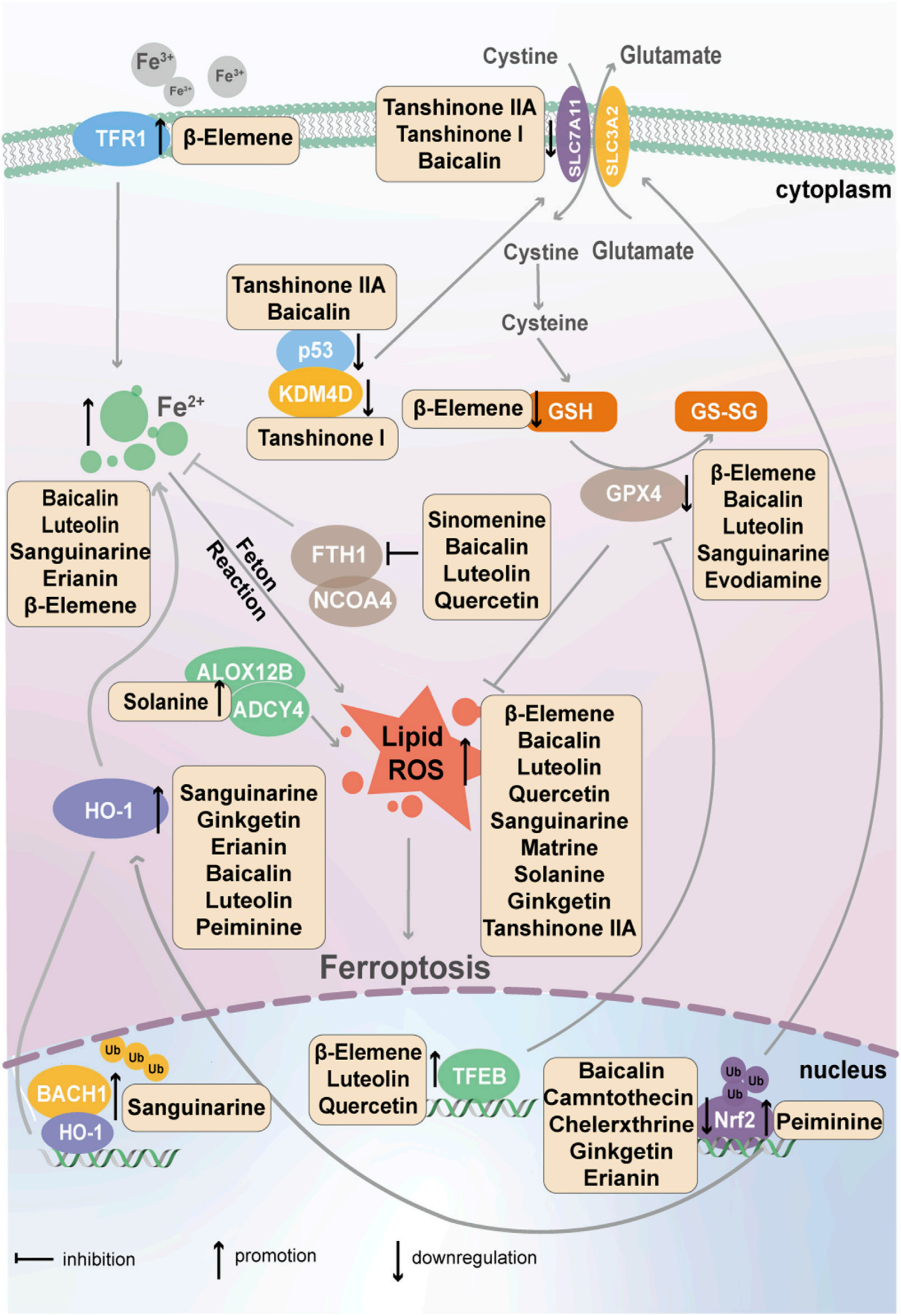
Natural active ingredients from plants, such as flavonoids, terpenoids and alkaloids, target the ferroptosis of cancer cells via transferrin, SLC7A11 and GPX4.

Ferroptosis is a newly discovered type of PCD, first proposed by Dixon et al., in 2012; its mechanism is related mainly to iron metabolism, which is mediated by transferrin; the amino acid antioxidant system, which is mediated by SLC7A11; and lipid peroxides, which are mediated by GPX4 (Zhang C. et al., 2022). The active ingredients in natural plants that target tumor cell ferroptosis are shown in Figure 3.

#### 2.4.1 Terpenoids

##### 2.4.1.1 β-Elemene

Studies have shown that β-Elemene can directly bind to transcription factor EB (TFEB), a master modulator of lysosome activation, and stimulate its activation and nuclear translocation, which in turn leads to GPX4 lysosomal degradation in NSCLC. As a key player in ferroptosis, GPX4 reduction promotes ferroptosis in cells, accompanied by the generation of massive amounts of ROS (Zhao et al., 2023). Moreover, β-Elemene enhances the sensitivity of many targeted drugs by triggering ferroptosis. Xu et al. showed that the combination of β-Elemene and erlotinib induces ferroptosis in EGFR-mutant NSCLC cells by promoting the expression of the lncRNA H19; however, the regulatory mechanism between lncRNA H19 and ferroptosis is unclear (Xu et al., 2023). In CRC, β-Elemene combined with cetuximab induces iron-dependent ROS accumulation, GSH depletion, the upregulation of transferrin expression, and lipid peroxidation, thereby resulting in ferroptosis (Chen et al., 2020a).

##### 2.4.1.2 Tanshinones I and tanshinone IIA

In gastric cancer, molecular docking revealed lysine-specific demethylase 4D (KDM4D) as the target of tanshinone I; this interaction reduces the protein level of KDM4D, which directly binds to p53 and suppresses its transcriptional activation of the downstream gene SLC7A11, ultimately leading to ferroptosis (Xia et al., 2023). Additionally, in gastric cancer, tanshinone IIA significantly activates lipid peroxidation and downregulates the expression of SLC7A11 by regulating p53 (Guan et al., 2020), leading to ferroptosis and decreased cell stemness (Ni et al., 2022).

In breast cancer, tanshinone IIA induces ferroptosis by reducing the SUMOylation of SLC7A11 and increasing its ubiquitin-mediated degradation. Further studies revealed that tanshinone IIA inhibits the expression of lysine-specific demethylase 1A (KDM1A), which epigenetically activates protein inhibitor of activated STAT4 (PIAS4) transcription, decreases the direct interaction between PIAS4 and SLC7A11 and inhibits SLC7A11 SUMOylation (Luo et al., 2024).

#### 2.4.2 Flavonoids

##### 2.4.2.1 Baicalin

Many investigations have revealed that baicalin can facilitate the anticancer effects of chemotherapeutic drugs through ferroptosis. For example, baicalin increases the sensitivity of gastric cancer cells to 5-fluorouracil through ROS-mediated ferroptosis (Yuan et al., 2023) and the sensitivity of gastric cancer cells to oxaliplatin by inducing ferroptosis via the p53/SLC7A11 pathway (Shao et al., 2024). In bladder cancer and oral squamous cell carcinoma, baicalin targets the key protein of ferroptosis, ferritin heavy chain 1 (FTH1), inhibits its expression and induces ferroptosis (Wen et al., 2024; Kong et al., 2021). Nrf2 is a transcription factor that stimulates the expression of many cytoprotective genes, such as heme oxygenase-1 (HO-1), to control the cellular oxidative stress response; however, HO-1 has dual roles in ferroptosis. In osteosarcoma cells, baicalin binds Nrf2 and promotes its ubiquitin-mediated degradation, leading to the downregulation of SLC7A11 and GPX4 expression and triggering ferroptosis (Wen RJ. et al., 2023).

##### 2.4.2.2 Luteolin

As a commonly used natural medicine, luteolin has been studied in the field of ferroptosis. In clear cell renal cell carcinoma, luteolin may promote the accumulation of unstable iron and heme degradation by directly binding HO-1 and stabilizing it, leading to the Fenton reaction and lipid peroxidation, thereby inducing ferroptosis (Han et al., 2022). Luteolin can also increase ferroptosis by regulating TFEB, increasing TFEB expression and nuclear translocation and subsequent FTH1 lysosomal degradation in prostate cancer (Fu et al., 2024). In colon cancer, luteolin combined with erastin targets the tumor suppressor hypermethylated in cancer 1 (HIC1) gene, which inhibits GPX4 expression, thereby inducing ferroptosis; however, HIC1 regulation underlying ferroptosis remains unclear (Zheng et al., 2023).

##### 2.4.2.3 Quercetin

Like luteolin, quercetin activates TFEB to cause FTH1 lysosomal degradation, thereby inducing ferroptosis in HCC, CRC and breast cancer cells. In addition, the generation of ROS caused by quercetin synergistically leads to lipid peroxidation and promotes ferroptosis (Wang ZX. et al., 2021; An and Hu, 2022).

#### 2.4.3 Alkaloids

##### 2.4.3.1 Sanguinarine

Research on natural alkaloid medicines and ferroptosis has increased annually. Sanguinarine, a classic representative of alkaloid drugs, has been widely used in cancer treatment. Ferroptosis can be induced by ROS production, a widely studied phenomenon. ROS, especially H_2_O_2_, reduce SLC7A11 and GPX4 expression in cervical cancer and prostate cancer (Liu S. et al., 2024; Alakkal et al., 2022); additionally, ROS stimulate BTB domain and CNC homolog 1 (BACH1) degradation by decreasing the expression of ubiquitin carboxyl-terminal hydrolase 47 (USP47), which can deubiquitinate BACH1. BACH1 binds to the HO-1 enhancer and suppresses its transcription; therefore, sanguinarine upregulates HO-1 expression, causing intracellular iron overload and ultimately inducing ferroptosis (Liu S. et al., 2024). In addition, sanguinarine facilitates the ubiquitin-mediated degradation of GPX4 through the E3 ligase STIP1 homology and U box-containing protein 1 (STUB1) in NSCLC cells and triggers ferroptosis (Xu R. et al., 2022).

##### 2.4.3.2 Camptothecin, chelerythrine, peiminine and matrine

Some studies have reported that camptothecin, peiminine, and chelerythrine target Nrf2 and promote ferroptosis. Camptothecin and chelerythrine have been shown to reduce Nrf2 expression in HCC and ovarian cancer, respectively (Zhou et al., 2024; Elkateb et al., 2023). In contrast, peiminine increases Nrf2 and downstream HO-1 expression in breast cancer (Yi et al., 2024). Intriguingly, matrine triggers ferroptosis in cervical cancer via piezo1 but has no effect on SLC7A11 or the transferrin receptor (TFR). The activated piezo1 channel enhances the influx of Ca^2+^, resulting in ROS generation and ferroptosis (Jin et al., 2024).

##### 2.4.3.3 Solanine, sinomenine and evodiamine

Solanine promotes the expression of arachidonate 12-lipoxygenase, 12R-type (ALOX12B) by enhancing its interaction with ADCY4, which stabilizes ALOX12B, leading to lipid peroxidation and ferroptosis in CRC (Ma et al., 2024). Sinomenine is an active alkaloid from *Sinomenium acutum*, and its derivative facilitates interactions between nuclear receptor coactivator 4 (NCOA4) and FTH1, accelerating Fe^2+^ release and ultimately promoting ferroptosis in colon cancer cells (Zhu et al., 2024). Yu et al. revealed that evodiamine can induce ferroptosis in prostate cancer through the upregulation of GPX4 expression (Yu et al., 2023).

#### 2.4.4 Other natural compounds

Lou et al. reported that ginkgetin enhances the anticancer effect of cisplatin and augments the unstable iron pool and lipid peroxidation, two hallmarks of ferroptosis, via Nrf2/HO-1 signaling inactivation in NSCLC (Lou et al., 2021). Erianin, a natural dibenzyl compound extracted from *Dendrobium chrysotoxum* (Dong et al., 2020), has also been found to downregulate Nrf2 and HO-1 expression to induce ferroptosis in bladder cancer (Xiang Y. et al., 2021). In lung cancer, erianin targets calmodulin and promotes its expression, and elevated calmodulin level facilitate Ca^2+^ and Fe^2+^ uptake, causing ferroptosis (Chen et al., 2020b).

### 2.5 Autophagy

**FIGURE 4:**
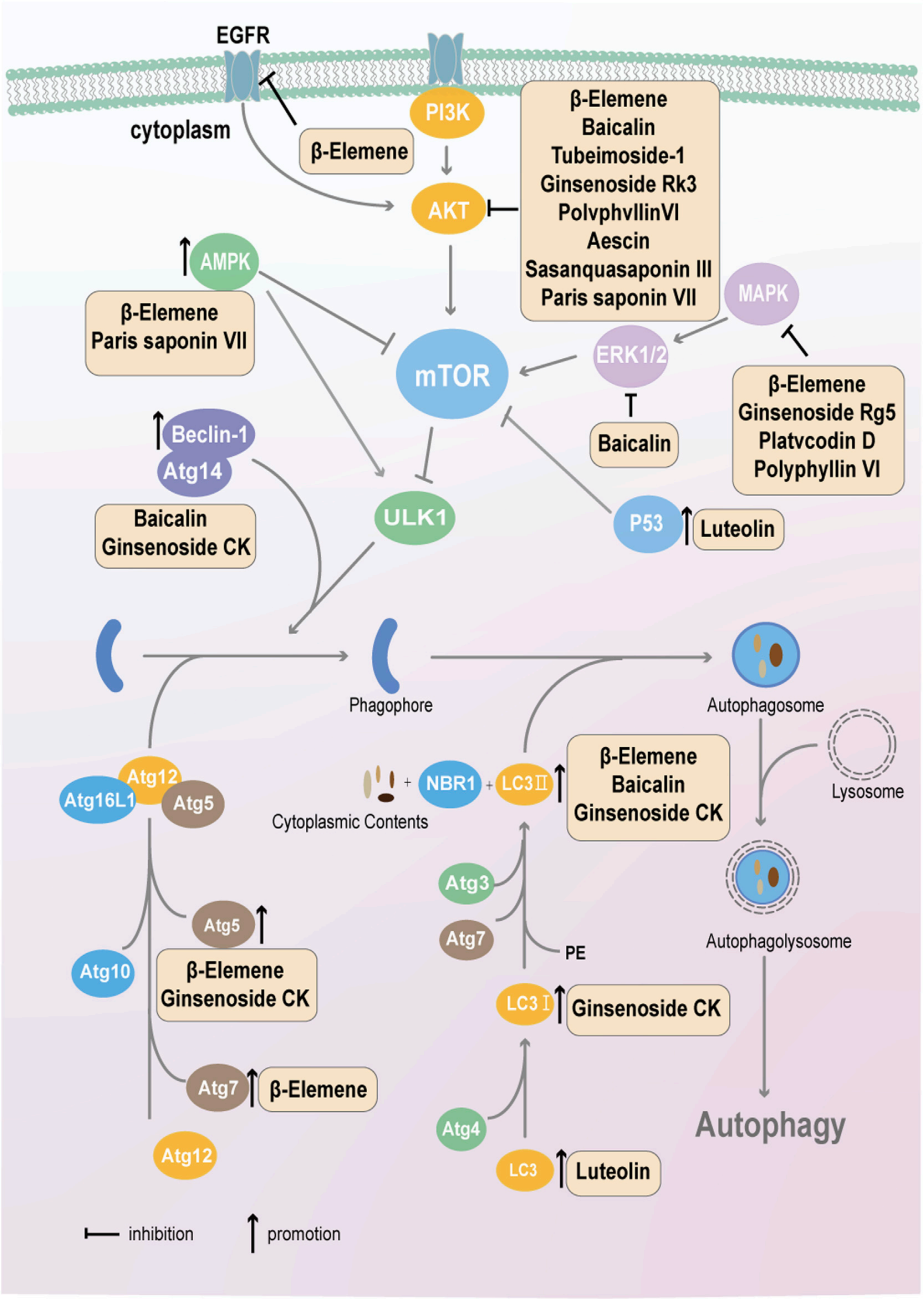
Natural active components from plants, such as flavonoids, terpenoids and saponins, target autophagy in cancer cells via the mTOR-ULK1-autophagosome pathway.

Autophagy is a process in which cells, under the fine regulation of a series of genes, degrade their own damaged organelles and macromolecules in lysosomes to achieve metabolic needs and the renewal of certain organelles (Kim and Lee, 2014). The natural plant compounds that target tumor cell autophagy are shown in Figure 4.

#### 2.5.1 Terpenoids

##### 2.5.1.1 β-Elemene

β-Elemene plays a role in promoting autophagy in cancer cells; it significantly promotes the conversion of LC3Ⅰ to LC3Ⅱ and then the formation of autophagosomes to promote autophagy in breast cancer and Ewing sarcoma (Guan et al., 2014; Zhang T. et al., 2023). In addition to directly targeting biomarkers of autophagy, β-Elemene also acts on upstream pathways, such as the PI3K/AKT/mTOR, MAPK/ERK, AMPK, and EGFR/AKT pathways, to control autophagy. In NSCLC, gastric cancer and renal cell carcinoma, β-Elemene significantly suppresses the activation of the PI3K/AKT/mTOR pathway, and in renal cell carcinoma, it also inhibits the MAPK/ERK pathway, thereby promoting the autophagy of cancer cells (Liu et al., 2012a; Zhan et al., 2012a; Liu et al., 2011). Furthermore, β-Elemene stimulates AMPK, thereby reducing the p-mTOR/mTOR ratio and ultimately causing autophagy in CRC cells (Wang et al., 2022).

A β-Elemene isopropanolamine derivative reduces the expression of 6-phosphofructo-2-kinase/fructose-2,6-bisphosphatase 4 (PFKFB4), a key enzyme in glycolysis, thus inhibiting glycolysis and triggering autophagy in melanoma cells (Jalal et al., 2022). β-Elemene sensitizes cells to chemotherapy drugs by targeting autophagy. For example, in NSCLC, β-Elemene enhances the effect of gefitinib by inducing autophagy; mechanistically, it decreases the protein level of the m^6^A methyltransferase METTL3 and the m^6^A methylation level in resistant cells, downregulates the expression of ATG5 and ATG7, which are critical components of the autophagy pathway, and ultimately inhibits autophagy (Liu et al., 2020e). β-Elemene also reverses resistance to gefitinib in GBM through the inactivation of the EGFR/AKT signaling pathway and activation of autophagy and enhances the anticancer effect of 5-fluorouracil in CRC by triggering autophagy; however, the detailed mechanism remains unknown (Mu et al., 2016; Zhang et al., 2020d).

#### 2.5.2 Flavonoids

##### 2.5.2.1 Baicalin

Baicalin might induce autophagy in osteosarcoma cells by directly binding to PI3Kγ and inhibiting the PI3K/AKT/mTOR, ERK1/2 and β-catenin signaling pathways (Pang et al., 2022a). Similarly, in bladder cancer, autophagy is achieved by inhibiting AKT expression and activity (Lin et al., 2013a). Additionally, baicalin can trigger autophagy in HCC cells and upregulate CD147 expression, which increases Beclin one expression through the activation of the PI3K/AKT pathway (Zhang et al., 2012a; Gou et al., 2009a). However, in nasopharyngeal carcinoma, baicalin downregulates LC3Ⅱ and Beclin 1 expression and inhibits autophagy, thereby reversing radioresistance (Wang et al., 2020).

##### 2.5.2.2 Luteolin

The role of luteolin in cancer autophagy may be controversial; even in one GBM type, luteolin has been reported to have dual functions in autophagy. Chakrabarti et al. reported that luteolin inhibits the expression of protein kinase C alpha type (PKCα) and autophagy (Chakrabarti and Ray, 2016); however, Lee et al. reported that it triggers autophagy and promotes cell survival but induces apoptosis to kill cells (Lee HS. et al., 2021). In liver cancer, luteolin stimulates autophagic flux, and one study reported that luteolin enhances cell viability (Lee and Kwon, 2019), whereas another study reported that it leads to TRAIL-induced apoptosis and cell death, possibly due to the use of distinct cell lines (Nazim and Park, 2019a).

There are also reports that luteolin promotes autophagy via p53; however, activated autophagy has no effect on the apoptosis or growth of colon cancer cells (Yoo et al., 2022). In TNBC, RNA sequencing and molecular docking demonstrated that luteolin targets serum and glucocorticoid-induced protein kinase 1 (SGK1) and inhibits its expression. The downregulation of SGK1 expression leads to decreased phosphorylation and the increased stability of the transcription factor forkhead box O 3a (FOXO3a), which activates the expression of bcl2/adenovirus e1b-interacting protein 3 (BNIP3), an inducer of autophagy through interactions with LC3 (Wu et al., 2023). Moreover, luteolin may increase cisplatin sensitivity by inhibiting autophagy in ovarian cancer (Liu et al., 2018). In summary, the regulatory effect of luteolin on cancer autophagy needs to be viewed objectively on the basis of different types of cancer and cell lines.

#### 2.5.3 Saponins

##### 2.5.3.1 Tubeimoside-1, ginsenoside Rk3, polyphyllin VI, aescin, sasanquasaponin ΙΙΙ, ginsenoside Rg5 and platycodin D

Saponins exert their anticancer effects mainly by suppressing cell proliferation and migration, inducing autophagy and promoting cancer cell apoptosis. In terms of mechanism, many reports have shown that saponin natural products target the upstream signaling pathway of autophagy. Tubeimoside-1 in breast cancer, ginsenoside Rk3 in HCC, polyphyllin VI in NSCLC, aescin in lung cancer, and sasanquasaponin ΙΙΙ in melanoma mainly target the PI3K/AKT/mTOR signaling pathway to induce cell autophagy; autophagy activated by tubeimoside-1 protects cells from death (Jiang et al., 2019), whereas the other four compounds result in cell death (Qu et al., 2023; Singh et al., 2023; Liang et al., 2019; Teng et al., 2019). The MAPK pathway also plays a vital role in cell autophagy. Research has revealed that ginsenoside Rg5 in gastric cancer, platycodin D in colon cancer, and polyphyllin VI in NSCLC promote cell autophagy and inhibit cell proliferation by inactivating the MAPK signaling pathway (Teng et al., 2019; Liu and Fan, 2019; Han et al., 2024).

##### 2.5.3.2 Paris saponin VII and ginsenoside CK

Paris saponin VII can activate the AMPK pathway, a signaling pathway that is opposite to AKT and MAPK, thereby inhibiting the mTOR signaling pathway and causing autophagy and cell death in NSCLC (Xiang YC. et al., 2021). In addition, Xiang et al. reported that Paris saponin VII directly interacts with the MST2-MOB1-LATS1 complex, increasing their interaction, thereby promoting the MOB1-mediated phosphorylation of LATS1 and LATS1-mediated phosphorylation and degradation of YAP, and that the inhibition of YAP ultimately activates the Hippo pathway and autophagy in breast cancer cells (Xiang et al., 2022). Ginsenoside CK, a saponin derivative, can trigger cell autophagy by downregulating the expression of SQSTM1/p62, a negative modulator of autophagy, and promoting the expression of Beclin-1, ATG5 and LC3-II (Yin et al., 2021).

### 2.6 The interrelationships between different PCD pathways in cancer cells

In recent years, it has been known that various PCD pathways can crosstalk with each other through regulatory signaling pathways and molecules. For instance, many studies have shown that ferroptosis plays an important role in the communications between different types of PCD. Iron overload is one of the causes of ferroptosis, it leads to the opening of the mitochondrial permeability transition pore, enhanced RIPK1 phosphorylation and ultimately necroptosis (Zhou et al., 2021). Ferroptosis and pyroptosis are antagonistic to each other, their interplay is regulated by cellular localization of 3-hydroxy-3-methylglutaryl-coenzyme A reductase (HMGCR) (Wang H. et al., 2024). Some natural plant compounds can simultaneously target multiple PCD pathways and lead to cancer cell death.

#### 2.6.1 Terpenoids

##### 2.6.1.1 β-Elemene

As mentioned above, β-Elemene can significantly inhibit PI3K/AKT/mTOR, MAPK pathways to induce apoptosis and autophagy, and combination of it with autophagy inhibitors can significantly enhance its anti-tumor effect (Liu et al., 2012b; Zhan et al., 2012b). Moreover, β-Elemene could reduce GSH synthesis and increase ROS to promote cancer cell apoptosis and ferroptosis (Chen et al., 2020a).

##### 2.6.1.2 Paclitaxel

Paclitaxel can increase PARP expression (Yu-Wei et al., 2020), and PARP is involved in TNF-induced necroptosis, therefore, upregulated PARP plays a pivotal role in promoting cancer cell apoptosis and necroptosis (Khing et al., 2019). Furthermore, the application of paclitaxel activates caspase-3/7 in multiple types of cancer cells, thereby inducing apoptosis and activating GSDME to cause pyroptosis (Zhang Y. et al., 2022).

#### 2.6.2 Flavonoids

##### 2.6.2.1 Luteolin

Luteolin simultaneously modulates cancer cell apoptosis and autophagy via two different mechanisms. First, it regulated p53 pathway and triggered apoptosis and autophagy of colon cancer cell (Yoo et al., 2022). Second, it activates JNK pathway and upregulates death receptor 5 to inducing apoptosis and autophagy (Nazim and Park, 2019b; Wu B. et al., 2020).

##### 2.6.2.2 Baicalin

Similar to β-Elemene, baicalin also triggers cancer cell apoptosis and autophagy by inhibiting the PI3K/AKT/mTOR pathway (Sui et al., 2020c; Pang et al., 2022b; Lin et al., 2013b; Zhang et al., 2012b; Gou et al., 2009b), but baicalin could also induce cell apoptosis via activating the AMPK pathway (Jia et al., 2022b).

##### 2.6.2.3 Apigenin

Apigenin treatment can lead to lots of ROS and induce apoptosis and necroptosis of cancer cells (Yang C. et al., 2021; Lee YJ. et al., 2020). In addition, it inhibits the PI3K/AKT/mTOR pathway to promote apoptosis and autophagy of HCC cells (Yang et al., 2018).

## 3 Conclusion

Cancer is a malignant disease caused by uncontrolled cell proliferation, and its pathogenic mechanisms are relatively complex and primarily involve the activation of oncogenes and the inactivation of tumor suppressor genes. In recent decades, the number of cancer cases and deaths has markedly increased annually; however, although the clinical management of cancer is constantly improving, the survival time and quality of life of cancer patients are still poor. Traditional Chinese medicines or a variety of monomeric herbal extracts, to a certain extent, strongly inhibit the occurrence and development of some cancers (Zou et al., 2024). Therefore, the use of active ingredients from plants to treat cancer has gradually attracted the interest of many researchers. These compounds are diverse and include terpenoids, alkaloids, flavonoids, etc. Using modern pharmaceutical technology to modify the formulation of these compounds enhances their anticancer effects; therefore, in addition to most herbal monomers, this review also introduces a few formulations containing monomers and their derivatives.

The antitumor mechanisms of these active ingredients are varied, and among these mechanisms, PCD is one of the most common and plays a pivotal role in the development of cancer. To date, there are many new research achievements on PCD in tumors. For instance, some groups have investigated the immunogenicity of zeolitic imidazolate framework-8 (ZIF-8) nanoparticles in tumor therapy and found that ZIF-8 nanoparticles can induce pyroptosis through the caspase-1/GSDMD pathway, leading to cancer cell necrosis, reprograming of the tumor microenvironment and activation of antitumor immunity (Ding et al., 2023). This research provides innovative ideas for the biomedical application of ZIF-8, and the combination of ZIF-8 with active plant ingredients that target cancer cell pyroptosis may show a synergistic effect. Similarly, studies have investigated the role of ferroptosis in tumor immunity. Drugs can induce ferroptosis in malignant cells but also simultaneously trigger ferroptosis in immune cells, which may compromise anti-tumor immunity. Interestingly, Li et al. found that the compound N6F11 could selectively induce GPX4 degradation and ferroptosis in pancreatic cancer cells and initiate CD8 T-cell anti-tumor immunity but had no impairment on immune cells. Mechanistically, N6F11 interacts with the E3 ubiquitin ligase TRIM25 and promotes the K48-linked ubiquitination and proteasomal degradation of GPX4 (Li J. et al., 2023). Immunotherapy is a popular treatment for cancer at present. It mainly includes immune checkpoint inhibitor (ICI) therapy, adoptive cell therapies (TILs, TCR-T and CAR-T) and tumor vaccines. However, the response rate of immunotherapy is still relatively low. To address this issue, the combination of immunotherapy with other drugs, for example, the combination of the commonly used PD-1/PD-L1 inhibitors and mature natural plant products, including luteolin, baicalin and β-Elemene (Lee J. et al., 2021), is a promising research direction.

Active ingredients from plants that target different forms of PCD can significantly suppress cancer cell viability or enhance the anticancer effect of chemotherapeutics and radiotherapy, ultimately inhibiting the occurrence and development of cancer, indicating that they have great potential as antitumor drugs or can be combined with other management strategies to synergistically increase their effectiveness. In summary, cell apoptosis, autophagy and ferroptosis are the main forms of PCD targeted by plant natural compounds, whereas other forms of PCD, such as necroptosis and pyroptosis, have been less studied. Specifically, these drugs directly regulate key components in different PCD pathways or modulate their upstream signaling pathways to control PCD. All of these findings provide a reference for the pharmacological study of newly discovered plant-derived antitumor drugs.

Recently, RNA sequencing and molecular docking have accelerated the mechanistic investigation of natural antitumor drugs (Zhang J. et al., 2022); however, the specific mechanisms of many compounds are still unknown. Metabolomics is widely used in basic tumor research. Tumor metabolism and metabolites, such as lactate and lactylation, are hot topics in tumor research. Thus, metabolomics can be applied to the study of antitumor drugs, which may help us find targets and deepen and broaden our research into the mechanisms of antitumor drugs. For example, it is worth studying and exploring whether drugs can alter lactate metabolism, regulate the lactylation of key molecules in the PCD pathway, and subsequently cause cell death. In recent years, studies have shown that mitochondrial dysfunction can cause TCA cycle failure, leakage of the electronic respiratory chain and the production of ROS, which ultimately leads to abnormal signal transduction and cancer. Therefore, mitochondria have become a new target for the treatment of cancer, and many plant compounds have been developed to target mitochondria. For example, matrine has been shown to activate mitochondrial fission through the Mst1-JNK pathway, leading to mitochondrial dysfunction, oxidative stress and cell apoptosis, thereby exerting anti-liver cancer effects (Cao et al., 2019). Apomorphine can induce mitochondrial depolarization, calcium overload and energy deprivation to trigger the apoptosis of human choriocarcinoma cells (Lee JY. et al., 2020). Furthermore, another study revealed that apomorphine could significantly inhibit lipid peroxides and ferroptosis (Miyauchi et al., 2024). However, this research was conducted in fibroblasts, and whether apomorphine has the same effect in cancer needs to be validated.

In the development of natural plant products, due to their large numbers of targets, their transformation into clinical drugs are limited to a certain extent. Moreover, the yields of some extracted plant compounds are very low, making it difficult to meet market demands. Although some natural products have therapeutic activities, the high incidence of their toxic side effects limit their use. Furthermore, many compounds are potential candidate drugs for cancer treatment by regulating PCD, most of them are currently based on experiments in cells and mice, and there is a lack of clinical research evaluating the clinical effects of such compounds. Since natural active ingredients from plants can be effective potential drugs for clinical cancer treatment, in the future, more such compounds should be identified to expand the candidate drug pool. Furthermore, the modification and biosynthesis of these compounds or their formulations to achieve the requirements of clinical trials is also needed, and more attention should be given to these compounds in clinical trials so that they can be used to treat cancer patients and improve their clinical efficacy.
